# se-atlas.de – Versorgungsatlas für Menschen mit Seltenen Erkrankungen

**DOI:** 10.1007/s00108-021-01085-y

**Published:** 2021-07-20

**Authors:** Michaela Neff, Jannik Schaaf, Niels Tegtbauer, Johanna Schäfer, Manuela Till, Thomas O. F. Wagner, Holm Graeßner, Christine Mundlos, Holger Storf

**Affiliations:** 1 Medical Informatics Group, Universitätsklinikum Frankfurt, Goethe Universität Frankfurt, Frankfurt am Main, Deutschland; 2 Frankfurter Referenzzentrum für Seltene Erkrankungen (FRZSE) und ERN-LUNG, Universitätsklinikum Frankfurt, Frankfurt am Main, Deutschland; 3 Institut für Medizinische Genetik und Angewandte Genomik, Zentrum für Seltene Erkrankungen Tübingen und ERN-RND, Universitätsklinikum Tübingen, Tübingen, Deutschland; 4 ACHSE e.V., c/o DRK Kliniken Berlin Mitte, Berlin, Deutschland

**Keywords:** Seltene Erkrankungen, Versorgungszentren, Patientenportale, Gesundheitspersonal, Gesundheitsversorgungseinrichtungen, Rare diseases, Care centers, Patient portals, Health personnel, Health facilities

## Abstract

Eine Erkrankung zählt in der Europäischen Union zu den Seltenen Erkrankungen (SE), wenn diese nicht mehr als 5 von 10.000 Menschen betrifft. Derzeit existiert mit mehr als 6000 SE eine sowohl große als auch heterogene Menge an unterschiedlichen Krankheitsbilder, die in ihrer Symptomatik komplex, vielschichtig und damit im medizinischen Alltag schwierig einzuordnen sind. Dies erschwert Diagnosefindung und Behandlung sowie das Auffinden eines passenden Ansprechpartners, da es nur wenige Experten für jede einzelne SE gibt. Der medizinische Versorgungsatlas für Seltene Erkrankungen www.se-atlas.de ermöglicht anhand von Erkrankungsnamen die Suche nach Versorgungseinrichtungen und Selbsthilfeorganisationen zu bestimmten SE und stellt die Suchergebnisse geografisch dar. Ebenso gibt er einen Überblick über alle deutschen Zentren für SE, die eine Anlaufstelle für betroffene Personen mit unklarer Diagnose darstellen. Der se-atlas dient als Kompass durch die heterogene Menge an Informationen über Versorgungseinrichtungen für SE und stellt niederschwellig Informationen für eine breite Nutzergruppe von Betroffenen bis hin zu Mitgliedern des medizinischen Versorgungsteams bereit.

## Abstract (en)

In the European Union a disease is classified as rare if it affects no more than 5 out of 10,000 people. Currently, there are more than 6000 rare diseases, consisting of a large and heterogeneous number of different diseases that are complex in their symptomatology, multidimensional and therefore difficult to classify in everyday medical practice. This complicates the diagnosis and treatment as well as finding a suitable contact person, as there are only a few experts for each individual rare disease. The medical care atlas for rare diseases www.se-atlas.de enables the search for care facilities and patient organizations for specific rare diseases by disease name and presents the search results geographically. It also provides an overview of all German centers for rare diseases, which are a contact point for patients with an unclear diagnosis. The se-atlas serves as a compass through the heterogeneous amount of information on care facilities for rare diseases and provides low-threshold information for a broad user group, from affected persons to members of the medical care team.

## Einleitung

Eine Erkrankung zählt in der Europäischen Union (EU) zu den Seltenen Erkrankungen (SE), wenn diese nicht mehr als 5 von 10.000 Menschen betrifft [2, 4]. Mit 3–6 % Betroffenen in der Gesamtbevölkerung entspricht dies deutschlandweit 2,4–5 Mio. Personen. Die Erkrankungsgruppe ist heterogen, und die einzelnen Erkrankungen unterscheiden sich oftmals in der Symptomatik nicht von häufigen Erkrankungen [8, 9, 16]. Webportale, wie der se-atlas, können dabei unterstützen, Informationen zu bündeln und den Zeitraum bis zu Diagnose und Therapie zu verkürzen.

## Hintergrund

Auf dem Weg zur Diagnose von SE beklagen Betroffene oft eine regelrechte „Odyssee“ von Arzt zu Arzt, ohne eine konkrete Diagnose zu erhalten. Betroffene müssen häufig lange Wegstrecken zurücklegen, um die richtige Diagnose und eine adäquate Therapie zu erhalten, da wenige Spezialisten und Versorgungseinrichtungen vorhanden und diese überregional verteilt sind [2, 3, 16]. Neben den Anlaufstellen der medizinischen Versorgung selbst sind die Informationen zu SE weit gestreut. Diese Problematik wurde durch das Nationale Aktionsbündnis für Menschen mit Seltenen Erkrankungen (NAMSE) in einem Aktionsplan adressiert. Eine Empfehlung des NAMSE war die Kartierung von Versorgungsangeboten für Menschen mit SE [5, 6]. Diese Empfehlung gab 2013 den Anstoß zur Entwicklung des medizinischen Versorgungsatlas für Seltene Erkrankungen, www.se-atlas.de, der einschließlich der zweiten Förderperiode bis Ende 2017 vom Bundesministerium für Gesundheit gefördert wurde [7]. Darauffolgend wurde der se-atlas mit geringem Aufwand gepflegt und weiterentwickelt. Eine langfristige Finanzierung im Rahmen der Finanzierung für Seltene Erkrankungen wird angestrebt.

Der www.se-atlas.de kartiert Versorgungsangebote für Menschen mit Seltenen Erkrankungen

Im Rahmen der Förderperiode wurde im ersten Schritt ein Prototyp entwickelt. Dieser wurde genutzt, um ein frühes Feedback der Zielgruppe (Betroffene und Personen aus dem Gesundheitsbereich) einzuholen. Bei der Entwicklung war den Teammitgliedern wichtig, die Expertise der Betroffenen und Patientenorganisationen hinsichtlich krankheitsspezifischer Versorgungseinrichtungen einzubinden. Wichtige Themen des Entwicklungsprozesses waren die Suche von Erkrankungen, die Darstellung der Einträge, die Zuordnung von einzelnen Erkrankungen zu Einrichtungen sowie das Qualitätsmanagement [14].

Zum Tag der SE am 28.02.2015 wurde der se-atlas erstmals veröffentlicht [7]. Mit mehr als 350.000 Aufrufen bis heute ist das öffentliche Interesse groß und wächst stetig (2015: 25.000 Aufrufe/Jahr, 2019/2020: 80.000 Aufrufe/Jahr). Der se-atlas stellt insbesondere für Betroffene und Mitglieder des medizinischen Versorgungsteams eine wichtige Informationsquelle dar. Die kontinuierlich wachsende Datenbasis ist ein entscheidender Faktor für die Akzeptanz bei der Zielgruppe [14]. Der Versorgungsatlas enthält 1808 Versorgungsangebote, 1012 Versorgungseinrichtungen, 207 übergeordnete Einrichtungen, wie Zentren für Seltene Erkrankungen (ZSE), und 302 Selbsthilfegruppen für SE (Stand März 2021).

Aufgrund der stetigen Nutzung und der Veränderungen der Anforderungen an Webseiten und Gesundheitsportale (z. B. steigende Nutzung von mobilen Endgeräten) wurde 2020 damit begonnen, den se-atlas umfassend zu überarbeiten. Da der optische Eindruck und die einfache Gestaltung einer Webseite einen signifikanten Einfluss auf das Nutzerverhalten haben [10], wurde auch dieser Aspekt berücksichtigt. Bei der Überarbeitung wurde – mit Blick auf die mobile Nutzung – auf eine einfache Bedienung sowie eine ansprechende, übersichtliche Oberfläche geachtet. Weitere Funktionen, wie beispielsweise die Suchfunktion, wurden verbessert. Die Veröffentlichung der neuen Version des se-atlas fand unmittelbar vor dem Tag der SE im Jahr 2021 (28.02.2021) statt – 6 Jahre nach dem Start der Webseite.

Der vorliegende Beitrag thematisiert die Datengrundlage und den Aufbau der neuesten, verfügbaren Version des se-atlas. Zudem wird beschrieben, wie der se-atlas aus unterschiedlichen Benutzersichten verwendet werden kann. Weiterhin wird ein Ausblick auf weiterführende und zukünftige Aktivitäten gegeben.

## Datengrundlage und Datendarstellung

### Datengrundlage und Qualitätsmanagement

Die Datenbasis des se-atlas baut auf verschiedenen Säulen auf. Dazu gehören neben Empfehlungen von Selbsthilfeorganisationen auch qualitätsgeprüfte Sammlungen von Orphanet und der Allianz Chronischer Seltener Erkrankungen e. V. (ACHSE e. V.) sowie von Fachgesellschaften für SE-zertifizierte Einrichtungen [7]. Die ACHSE e. V. ist der deutsche Dachverband von und für Menschen mit chronischen SE und deren Angehörigen. Sie bündelt inzwischen mehr als 130 Patientenorganisationen sowie Expertise und Wissen im Bereich SE [1, 11]. Orphanet ist eine länderübergreifende Datenbank und bündelt Ressourcen zu SE und Medikamenten zu deren Behandlung [12]. Die Bestätigung der durch die unterschiedlichen Quellen bereitgestellten Daten (z. B. Angaben zu krankheitsspezifischen Versorgungseinrichtungen) erfolgt durch eine interne Prüfung oder auf Basis der Auskünfte von Selbsthilfeorganisationen. Darüber hinaus können Nutzende, die z. B. die Vertretung von Selbsthilfeorganisationen übernehmen, selbst Angaben zu Einrichtungen einreichen und eine Freischaltung durch die Redaktion beantragen. Das Redaktionsteam, das über medizinische Kenntnisse verfügt, prüft bei der Eintragsfreischaltung die Daten durch den Abgleich mit verschiedenen Quellen (z. B. Vergleich mit einer Zertifizierung durch eine Fachgesellschaft). Auch bestehende Einträge werden in einem kontinuierlichen Verbesserungsprozess regelmäßig überprüft. Ebenso haben Selbsthilfeorganisationen die Möglichkeit, Einrichtungen, deren zugeordnete Erkrankung sie vertreten, zu evaluieren [7, 15].

### Datendarstellung

**Figure 1:**
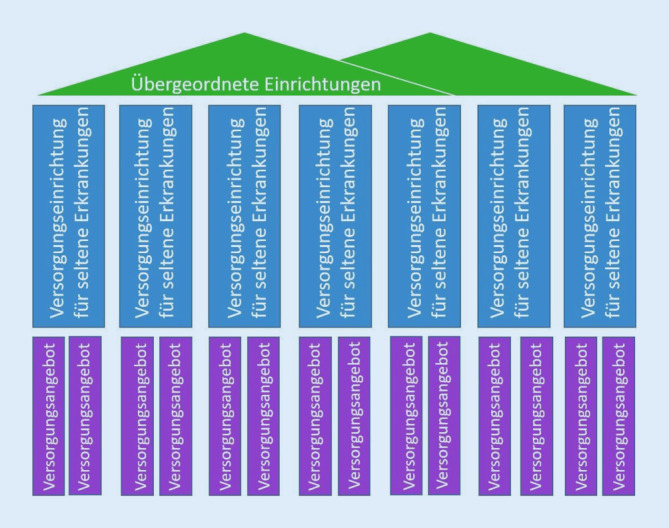

Der Grundaufbau der Einträge des se-atlas (Abb. 1) ist an das Zentrenmodell der NAMSE angelehnt. Das Dach der Einrichtungen bilden sog. Referenzzentren (Typ-A-Zentren). Diesen übergeordneten Zentren, zu denen die ZSE gehören, sind Versorgungseinrichtungen (Typ-B-Zentren) als Fachzentren für spezifische SE und Erkrankungsgruppen mit ihrem Versorgungsangebot untergeordnet. Beispielsweise sind dem Frankfurter Referenzzentrum für Seltene Erkrankungen mehrere Versorgungseinrichtungen untergeordnet, zu denen das Epilepsiezentrum Frankfurt Rhein-Main gehört. Das Epilepsiezentrum bietet mehrere spezielle Sprechstunden, z. B. eine zur tuberösen Sklerose, an. Das dreistufige Modell erlaubt es, die vorhandenen Klinikstrukturen in Deutschland abzubilden und somit stationäre und ambulante Angebote darzustellen [7]. Auch Institutionen für Genetik sind unter den Versorgungseinrichtungen gelistet.

## se-atlas im Anwendungskontext: Recherche mit dem Versorgungsatlas

**Figure 2:**
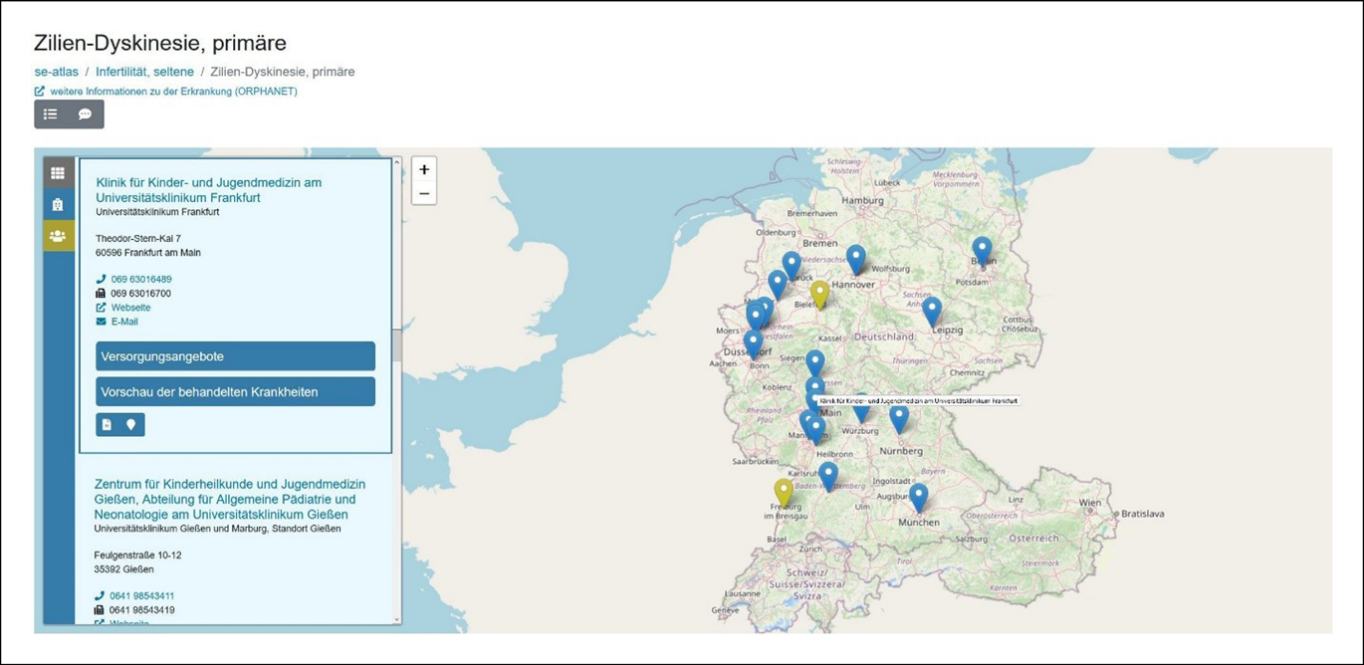

Die direkt auf der Startseite auffindbare Suche von Versorgungseinrichtungen und Selbsthilfeorganisationen stellt die zentrale Funktion des se-atlas dar. Mit ihr kann über die von Orphanet bereitgestellte und im se-atlas hinterlegte Klassifikation der SE eine Erkrankung gesucht werden, die mit Versorgungseinrichtungen und Selbsthilfeorganisationen verknüpft ist – wenn es hierfür in Deutschland ein Angebot gibt. Nach Eingabe von mindestens 3 Zeichen des Suchbegriffes erhält man Vorschläge, aus denen der gewünschte Erkrankungsname auswählt werden kann. Zugleich wird direkt ersichtlich, wie viele Versorgungseinrichtungen und Selbsthilfeorganisationen zu dieser Erkrankung hinterlegt sind. Die detaillierte Darstellung der Ergebnisse zum Suchterminus erfolgt in einer Listen- und einer Kartenansicht (Abb. 2).

Zusammenfassend bietet der se-atlas den Nutzenden neben der zentralen Suchfunktion folgende wichtige Funktionen:

- detaillierte Informationen zu einer bestimmten Einrichtung,
- Informationen zu ZSE,
- Informationen zu Netzwerken und zertifizierten Einrichtungen,
- allgemeine und spezifische Informationen im Bereich der SE.

Im Folgenden werden typische Anwendungsszenarien der genannten Funktionen anhand von 3 Beispielen erläutert.

### Fallbeispiel 1: Eine betroffene Person mit *ungeklärter *Diagnose sucht nach Ansprechpersonen.

**Figure 3:**
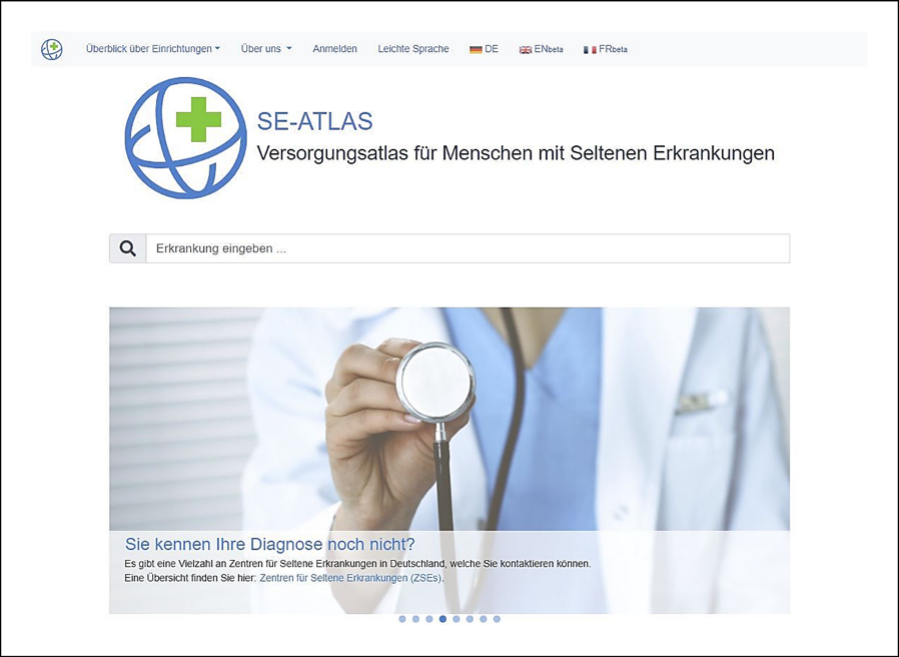

Eine betroffene Person konnte bisher keine Diagnose für ihr Leiden erhalten und stößt bei ihrer Suche nach Hilfe über eine Suchmaschine auf den se-atlas. Zuerst gelangt sie auf die Startseite. Hier soll es in leichter Form ermöglicht werden, eine erste Ansprechperson zu finden, indem man von der Startseite mit der Frage „Sie kennen Ihre Diagnose noch nicht?“ (Abb. 3) direkt auf die Übersicht der ZSE weitergeleitet wird. Die betroffene Person kann mithilfe einer Karte oder einer Liste das Zentrum suchen, das sich am nächsten zu ihrem Wohnort befindet und dieses kontaktieren.

Auf der Suche nach der Diagnose finden Betroffene erste Ansprechpersonen

**Figure 4:**
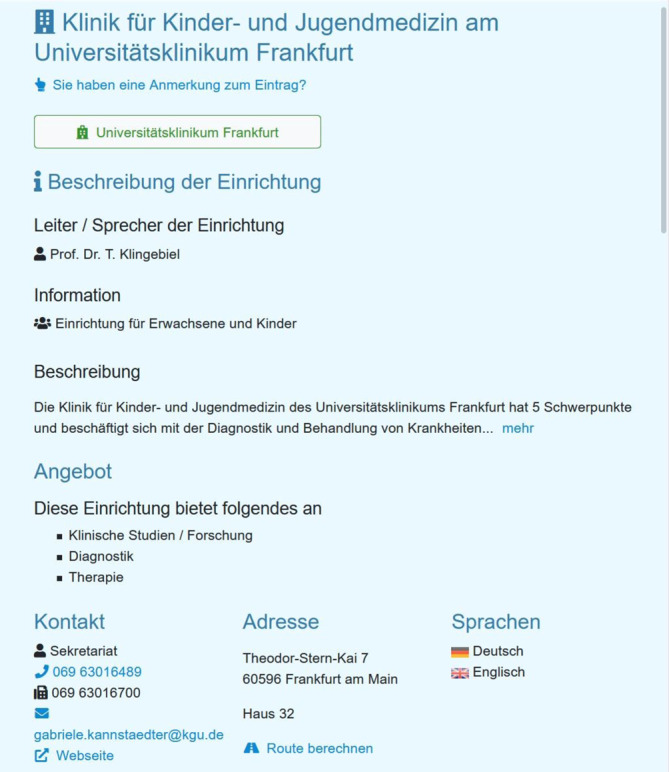

**Figure 5:**
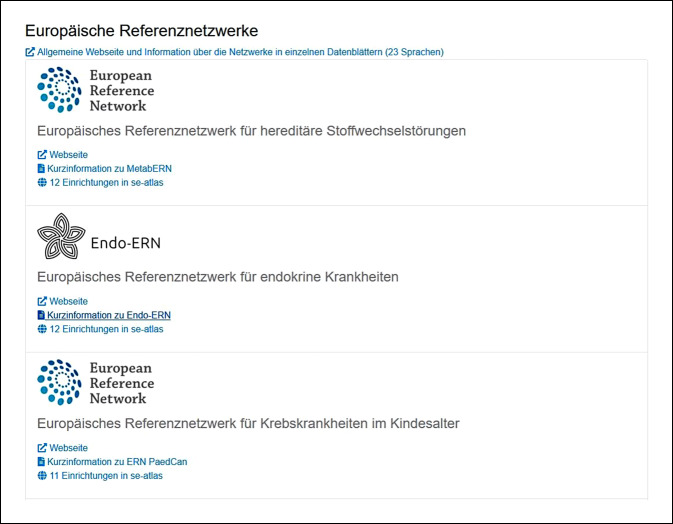

An den Zentren können der betroffenen Person die sog. Lotsen weiterhelfen oder sie weitervermitteln. Die detaillierte Darstellung der einzelnen Zentren und Einrichtungen (Abb. 4), auf die die betroffene Person durch Klick auf den Einrichtungsnamen gelangt, ermöglicht es, Details, beispielsweise über die Einrichtung selbst und die gesprochenen Sprachen vor Ort, einzusehen. Ebenfalls sind die einzelnen Versorgungsangebote auf dieser Detailansicht in einer Liste übersichtlich dargestellt, und der betroffenen Person werden die für ihre Erkrankung spezialisierten Angebote markiert. Diese Information kann neben dem geografischen Standort bei der Auswahl der Einrichtung wichtig sein. Einen weiteren Überblick im Bereich der SE können sich Betroffene auf den Übersichtsseiten der europäischen Referenznetzwerke (ERN) und der im se-atlas gelisteten zertifizierten Einrichtungen verschaffen (Abb. 5). Für die Suche nach Kontaktmöglichkeiten ist auch die ACHSE e. V. verlinkt, die eine persönliche Beratung anbietet und ein wichtiges Bindeglied darstellt.

### Fallbeispiel 2: Eine medizinische Fachkraft möchte eine betroffene Person an eine passende Einrichtung *weitervermitteln*

Eine medizinische Fachkraft betreut eine betroffene Person mit einer diagnostizierten SE oder einer noch nicht geklärten Diagnose mit Verdacht auf eine bestimmte SE. Die behandelnde Person möchte die betroffene Person an eine spezialisierte Einrichtung weitervermitteln, um beispielsweise eine weitere Meinung zu dem Fall einzuholen. Eine entsprechende Einrichtung mit Ansprechperson kann über die zentrale Suchfunktion bei Eingabe des Erkrankungsnamens gefunden werden. In der Kartenansicht (Abb. 2) kann anschließend die Einrichtungen in der Nähe herausgesucht und ihr Angebot genauer geprüft werden.

Wenn eine Einrichtung für die Person infrage kommt, findet sich auf der detaillierten Ansicht (Abb. 4) die entsprechende Ansprechperson und Kontaktoptionen. Auch in diesem Fall werden die Versorgungsangebote, die für die Person an diesem Standort spezialisiert sind, markiert. Als weitere Unterstützung kann die Fachkraft die betroffene Person an eine passende Selbsthilfeorganisation vermitteln, die sie gleichfalls über die Suche findet. Über den weiterleitenden Link zu Orphanet, der bei der Darstellung der gesuchten Erkrankung mitangezeigt wird, kann man sich auf der Webseite dieser Datenbank weiter über die Erkrankung informieren. Ebenfalls besteht die Möglichkeit, das nächstgelegene ZSE zu kontaktieren, beispielsweise zum fachlichen Austausch oder zur Weitervermittlung im Hinblick auf die Diagnosestellung.

Medizinische Fachkräfte finden Ansprechpartner und Kontaktoptionen zu spezialisierten Einrichtungen

### Fallbeispiel 3: Eine betroffene Person mit *bekannter* Diagnose sucht nach Kontaktdaten

Eine betroffene Person hat ihre Diagnose bereits erhalten, sucht jedoch nach weiteren Kontaktdaten, z. B. von Selbsthilfeorganisationen zum Austausch mit anderen Betroffenen. Sie kann direkt nach dem ihr bekannten Erkrankungsnamen suchen und über die detaillierte Darstellung ihrer Suchergebnisse mehr über die Einrichtungen erfahren (Abb. 4). Sie kann Selbsthilfeorganisationen kontaktieren und sich über die Spezialsprechstunden von Versorgungseinrichtungen informieren. Die Versorgungseinrichtungen verweisen immer auch auf ihre übergeordneten Einrichtungen.

**Figure 6:**
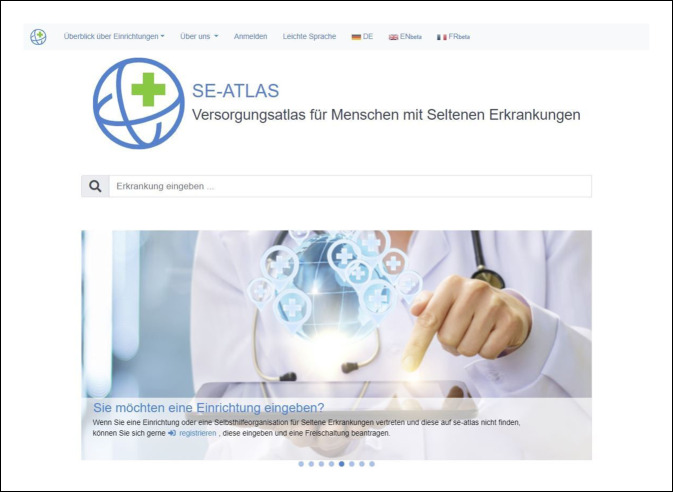

Wenn die suchende Person eine Selbsthilfeorganisation für eine bestimmte Erkrankung vertritt und diese im se-atlas nicht vorfindet, kann sie selbst aktiv werden (Abb. 6). Nach einer Registrierung kann sie, eingeloggt, Versorgungseinrichtungen und Selbsthilfeorganisationen mit allen Informationen wie Kontaktperson, Lokalisierung und Angebot als eigene Einträge anlegen. Nach der Fertigstellung kann eine Freigabe durch die Redaktion beantragt werden. Vertreter einer Selbsthilfeorganisation für eine spezifische Erkrankung oder Erkrankungsgruppe haben ebenfalls die Möglichkeit, krankheitsspezifische Einrichtungen zu beurteilen. Somit wird ein stetiger Informationszuwachs und Wissensaustausch für den se-atlas ermöglicht.

## Perspektiven des se-atlas

Der Datenbestand des se-atlas soll weiterhin stetig wachsen und das Versorgungsangebot von SE in Deutschland bestmöglich abbilden. Neben der Steigerung der Zahl der Einträge ist es wichtig, dass die Aktualisierung von allen Stakeholdern vorangetrieben wird. Nach wie vor wird angestrebt, alle Krankheitsgruppen der SE gut und qualitätsgesichert mit Versorgungseinrichtungen und -angeboten abzudecken. Zur Qualitätssicherung können Ergebnisse aus dem Verbundprojekt Collaboration on Rare Diseases (CORD) im Rahmen der Medizininformatik-Initiative (MII) herangezogen werden. In diesem nationalen Projekt arbeiten 20 Universitätskliniken mit vielen weiteren Projektbeteiligten zusammen, um digitale Informationen aus Diagnostik, Therapie und Forschung gemeinsam datenschutzkonform nutzen zu können und damit die Versorgung und Lebensqualität von Betroffenen mit SE zu verbessern. Die Ergebnisse von CORD sollen im se-atlas geografisch dargestellt werden. Ebenfalls kann mithilfe von Fallzahlen der teilnehmenden Krankenhäuser die Expertise der jeweiligen Versorgungseinrichtung validiert werden. Die Inhalte sollen des Weiteren unter anderem durch die Darstellung der deutschen Referenznetzwerke mit ihren zugehörigen Versorgungseinrichtungen und passenden Selbsthilfeorganisationen im se-atlas erweitert werden. Die Rückmeldungen von Selbsthilfeorganisationen bleiben von besonderer Bedeutung, da diese über ein fundiertes Wissen zu den von ihnen vertretenen Erkrankungen verfügen und einen guten Überblick über die Versorgungslandschaft auf dem speziellen Gebiet besitzen.

Weitere Ziele sind die regelmäßige Erweiterung von Verlinkungen zu relevanten Webseiten zum Thema SE sowie die stetige Weiterentwicklung der Benutzerfreundlichkeit. Des Weiteren ist es wichtig, die Reichweite des se-atlas zu vergrößern. Die Plattform sollte v. a. auch Personengruppen wie den Betroffenen mit ungeklärter Diagnose und ärztlichem Personal noch bekannter gemacht und die Auffindbarkeit über Suchmaschinen soll gesteigert werden.

In der Vergangenheit wurde der se-atlas bereits als Instrument zur Diagnoseunterstützung in ein klinisches Entscheidungsunterstützungssystem für SE integriert, dass im Rahmen eines Projekts des MIRACUM-Konsortiums (Medical Informatics in Research and Medicine) entwickelt wurde. Das System ermöglicht es, an den 10 teilnehmenden Universitätskliniken zu einem nichtdiagnostizierten Patientenfall einen ähnlichen Patientenfall zu finden. Liegt eine Verdachtsdiagnose vor, kann ein Experte für eine SE über eine Schnittstelle zum se-atlas kontaktiert werden [13].

In Zukunft kann an alle genannten Punkte und Projekte angeknüpft und so die Vielfältigkeit des Nutzens des se-atlas weitergesteigert werden.

## Fazit für die Praxis

- Seltene Erkrankungen (SE) bilden eine heterogene Gruppe von komplexen Krankheitsbildern mit besonderen Anforderungen an die Versorgung.
- Mithilfe des se-atlas kann der Zeitraum bis zur Diagnosestellung und zum Auffinden einer passenden Versorgungseinrichtung verkürzt werden.
- Die Zielgruppe des se-atlas reicht von Betroffenen über medizinisches Personal bis zum interessierten Internetnutzenden.
- Der se-atlas möchte Mitgliedern des medizinischen Versorgungsteams bei der Vermittlung und Vernetzung im Bereich der SE unterstützen.
- Der Datenbestand des se-atlas ist seit 2015 stetig gewachsen und soll in Zukunft weiterwachsen.
- Ein wichtiger Aspekt bei der Verbesserung und regelmäßigen Aktualisierung des se-atlas ist die aktive Mitgestaltung durch alle Stakeholder.

## Abkürzungen

CORD: Collaboration on Rare Diseases
EU: Europäischen Union
ERN: Europäische Referenznetzwerke
MII: Medizininformatik-Initiative
NAMSE: Nationales Aktionsbündnis für Menschen mit Seltenen Erkrankungen
SE: Seltene Erkrankungen
ZSE: Zentren für Seltene Erkrankungen
